# Hot Springs, South Dakota; a Health Resort

**Published:** 1895-04

**Authors:** J. W. Grosvenor

**Affiliations:** 118 Plymouth Avenue; Buffalo, N. Y.


Buffalo Medical and Surgical Journal

Vol. XXXIV. APRIL, 1895. No. 9.

### Original Communications.

### HOT SPRINGS, SOUTH DAKOTA; A HEALTH RESORT.

By J. W. GROSVENOR, M. D., Buffalo, N. Y.

Climates and natural waters have become important factors in the treatment of disease. The physician of today is expected to be well-posted on the various climates and health resorts of his own country and to have a general knowledge of them as they exist in foreign countries. In the not distant future the climatological specialist will have his office in every large city of the land, where he will be consulted by his clients and, for a consideration, will point out the best localities for the climatic cure of the various diseases with which he may come in contact. In this article it is my purpose to present to the medical profession some advantages of a young health resort which of late has found great favor with physicians of the West and Northwest.

Hot Springs, S. D., is situated in the southwestern part of the state, not far from the eastern border of Wyoming and at the beginning of the approaches to the Black Hills.

Its main site is the bottom of a gulch or cañon, thirteen miles in length and varying from a few hundred to many hundred feet in breadth. In some places the sides of the valley are quite precipitous; in others they ascend gradually into lofty hills. Opposite Hot Springs, on the top of the embankments, are broad plateaus, which furnish eligible sites for residences and public buildings.

Along this cañon for about six miles springs of warm water bubble up out of the earth in sufficient quantity to constitute a small stream of pure limpid water, which flows at a rather rapid rate through the center of the valley and is called Fall River.

Parallel to the river run a railroad and a carriage road and on either side of it are hotels, business blocks and public buildings.

*History.*—Less than a score of years ago the whole region, of which Hot Springs is the central point, was occupied by Indians. They had recognized—perhaps from time immemorial—the healing virtues of these spring-waters and had brought their sick there for bathing. Marvelous stories of the transforming powers of the waters have been handed down from one generation to another. They are more or less legendary in character. The cures that were wrought were observed by white men, and about fifteen years ago an enterprising physician, Dr. R. D. Jennings, purchased one of the springs, the Minnekahta, and its surrounding territory from the Indians and immediately began the use of the waters for his patients. The little hamlet of that day has grown into a city of 2,500 inhabitants.

*Analysis of the Waters.*—Although analysis of the many springs which are gushing up here and there in and near the valley shows that they differ somewhat in their ingredients, they are all essentially the same, the variations observed being caused by a slight difference in the soils through which they pass.

A characteristic of the waters of all the springs of this region is their temperature, which is nearly as high as that of the body, being from 94° to 96° Fahrenheit.

*Minnekahta Spring.*—This pleasantly-sounding name was given to it by the Indians and means hot water. The water of this spring has a higher temperature than that of any of the others. It is the spring used by the Indians and by which their aged were rejuvenated and their cripples were made "to leap as a hart." In the bath-house built over it is the ancient Indian bathing tub of immense proportions hewn out of the solid rock. Chemical analysis of this spring has shown its composition to be as follows:

| Constituents, per gallon. | Grains. |
|---|---|
| Total residue | 62.52 |
| Sodium sulphate | 6.11 |
| Potassium sulphate | 19.51 |
| Calcium sulphate | 16.33 |
| Sesqui-oxide of Iron | a trace |
| Silica | 2.46 |
| Magnesium sulphate | 4.32 |
| Sodium chloride | 13.79 |

Other important springs are the Mammoth, which furnishes the plunge bath-house and supplies the city with water; the Catholicon, which is connected with a hotel and bathing establishment of the same name; the Hygeia, which has the reputation of being specially adapted to the cure of kidney and urinary diseases.

The water furnished to the Stewart bath-house is supplied through wells sunk to the hot water vein. From the spring waters which rise to the surface it differs in composition and in having a somewhat higher temperature. Its composition is as follows:

| Constituents, per gallon. | Grains. |
|---|---|
| Total residue | 100.00 |
| Sulphate of sodium | 12.50 |
| Sulphate of potassium | 13.10 |
| Sulphate of calcium | 8.05 |
| Nitrate of magnesium | 1.90 |
| Iron sesqui-oxide | 4.00 |
| Silica | 4.01 |
| Sulphate of magnesium | 14.09 |
| Chloride of sodium | 14.00 |
| Potassium chlorate | 13.00 |
| Bromide and iodide of potassium | 15.35 |

*Rheumatism.*—This is the disease which preëminently has the reputation of being curable by the waters of these springs. The following is one of scores of cases which might be adduced to prove the truth of this assertion:

A man 45 years of age had had rheumatism for fourteen years; had been treated by many physicians and also had used the waters at Colfax Springs. For the last two years had been almost totally disabled. The disease affected all the large joints of the body, which were stiffened and greatly swollen. On his arrival at Hot Springs he was apparently a physical wreck. His weight was 106 pounds. In walking a few rods from the railroad station to the hotel he used two crutches, but was unable to move them without assistance. He drank the water freely and took daily baths. At the end of six weeks he was able to superintend a news stand. At the end of eight weeks he had gained twenty pounds; used only a cane in walking; had almost complete use of his arms; joints were still somewhat enlarged, but gradually growing smaller. From a positively helpless cripple he had been transformed into a competent man of business.

*Other Diseases.*—It is my earnest belief that these waters have a decidedly salutary effect upon diseases of the bladder and kidneys. Subjects of various kinds of skin diseases have here found their liberation from maladies disgusting and obstinate. Records of resident physicians show that derangements of the digestive system, both functional and organic, have received marked benefit from a proper use of these waters. By their use all conditions of the system in which there is an accumulation of noxious materials undergo a cleansing process. To secure its highest benefit the waters must be used both externally and internally. It is not at all disagreeable to the taste—indeed, is quite palatable. Taken in large quantities it is laxative, but not severely so.

From personal experience I can declare that a bath in these waters is a positive delight. They impart a smooth, oleaginous feeling to the skin, which I have never experienced to so large an extent by bathing in any other water. The dryness of the atmosphere encourages perspiration for a long time after leaving the bath and thus the body is enabled quickly to dispel the poisonous agents with which it may be freighted. Many invalids find benefit from drinking large quantities of the water. The resident physicians who understand its characteristics frequently direct their patients "to fill up on it three times a day."

*Climate.*—During the last eight years at Hot Springs a reliable private diary, in statistical form, has been kept concerning the climate. Dr. A. F. McKay, editor of *Climates and Resorts*, has recently compiled these statistics in such a way as to exhibit at a glance the prominent features of the climate. This exhibit shows the climate to be equable, mild, sunny and dry.

The situation of Hot Springs, in a narrow cañon, gives it freedom from both severe and sudden storms which at times affect the Dakotas. The surrounding hills protect it from the ravages of the cyclonic winds which sweep through some other regions of the state.

In general, it may be stated that the average daily temperature for the winter months will not freeze water. The snow-fall is light. Usually the snow falls during the night and melts the following day.

A sleigh ride in Hot Springs is a rare pastime. At one period, however, during the eight years the snow fell to the depth of ten inches and remained on the ground six days. The average number of days in which snow fell was: In October, one day; in November, two days; in December, three and one-half days; in January, three and one-half days; in February, eight and three-fourths days; in March, eight and three-fourths days. Although the nights may be cool or cold, on nearly every winter day a person is comfortable out-of-doors without an overcoat.

The heat in summer cannot be called excessive, the mercury seldom reaching the 100° limit and then only for a short period at mid-day.

Hot Springs is remarkably free from wind. Two and one-half is the average number of days per month on which there was sufficient wind to call for a note of its existence. There are no damp, chilling blasts and destructive blizzards.

So many are the sunny days, that truthfully it may be called a land of sunshine. The average number of perfectly clear days per month for eight years was twenty-one and one-half; of days partially clear, four and one-eighth; of cloudy days, four and seven-eighths. In 1893, the total number of cloudy days was thirty-three. A day was regarded as cloudy when during more than half of it the sun could not be seen. Surely such an exhibition of sunlight is entitled to high commendation.

The dryness of the atmosphere is shown by the average yearly amount of rainfall, which is seventeen inches.

But few health resorts in the country can show such a light rainfall. For the most part the rain falls in brief showers. In eight years there have been only a half dozen genuine rainy days. This record substantiates the claim that this region has a *dry* climate.

The altitude of Hot Springs, 3,400 feet above sea level, lifts it above the heavy atmosphere of lower levels and imparts to it a vigor which is grateful and life-giving to sufferers from certain kinds of pulmonary disease. It is not sufficiently elevated to produce an injurious effect upon the heart and general circulation. Invalids who cannot endure either very high or very low altitudes will find here verified the Latin adage, "*In medio tutissimus ibis.*"

Hot Springs is anti-malarial. Mountain fever is said to exist to some extent in surrounding regions rather remote, but seldom or never does a case of malaria occur in the city itself. It is even disputed, with a good deal of positiveness, that mountain fever is of malarial origin.

*Consumption and Asthma.*—From the above description of the climate it is not difficult to draw the conclusion that it has a favorable influence upon diseases of the lungs. This conclusion accords with experience and observation—especially in connection with consumption and asthma. The consumptive or asthmatic who can revel in the sunshine out-of-doors 306 days every year at a moderately high altitude without being subjected to sudden climatic changes, in a dry atmosphere and a mild temperature, must certainly receive great benefit from his environment.

*Neurasthenia.*—By one physician whom I interviewed, Dr. C. W. Hargens, Hot Springs is regarded as a most excellent resort for neurasthenics. This is probably due, in part, to the invigorating atmosphere, to the fact that they can spend a large part of the time in the open air and to the quietness of the city and its surroundings. It is generally acknowledged that out-door exercise and freedom from much excitement are conducive to the welfare of the neurasthenic. Quiet pleasures and amusements are easily found by those who need them.

*The Soldiers' Home.*—Not long since, about seventy veterans of the late civil war, suffering from a variety of ailments, were sent from the Soldiers' Home at Leavenworth, Kan., to Hot Springs to undergo treatment. They were enfeebled by old age as well as by disease. After spending a few months there all but two were cured and those two greatly benefited.

The attention of the military committee of congress was directed to this successful test, and Hot Springs was soon selected by that committee as the best locality in the United States for a Veterans' National Sanitarium.

The sanitarium building, constructed of the beautiful pink sandstone of this region and capable of accommodating 140 soldiers, has been completed. At the present time it affords a shelter and comfortable home to 125 veterans. Perhaps no higher recommendation can be given to the healthfulness of Hot Springs and its adaptability to the cure of disease than the presence of this Soldiers' National Sanitarium.

*The Plunge Bath.*—This bath-house is the largest of its kind in the country; its dimensions are 75 x 250 feet. One hundred thousand gallons of pure, clear, warm water run into it and out of it every hour. In it all ages and sizes of human beings, from the infant to the septuagenarian, may be seen swimming, diving and frolicking in every way conceivable. Every boy and girl should learn the art of swimming. The exercise is healthful and a knowledge of this art is of essential service in all cases of accident by water. The physician should regard it an imperative duty to advise the families under his professional care upon this important matter. No better place for learning to swim can be found than the Plunge at Hot Springs.

The Evans bath-house has been constructed in connection with the large and commodious hotel of the same name. It is under the careful supervision of a competent physician and is complete in all the appointments of a first-class bathing establishment.

Other advantages of this invalids' resort besides those mentioned, are the excellent accommodations at hotels and boarding-houses at moderate prices, the social refinement and high moral tone of the inhabitants, the magnificent scenery of mountains, valleys and streams, places of historic interest in the near surroundings, an atmosphere impregnated with the agreeable exhalations from the pine and spruce forests of the Black Hills.

*The Journey.*--Hot Springs is quite accessible by two routes from Chicago, from which it is distant from thirty-six to thirty-eight hours. The B. & M. R. route, a part of the Burlington system running to Deadwood, has a branch line to Hot Springs from Minnekahta, a distance of ten miles. Leaving Chicago at 6.30 in the evening the traveler reaches the Springs the second morning.

The F., E. & M. V. route, a part of the Chicago & Northwestern system, has a branch to Hot Springs from Buffalo Gap.

It was my good fortune on a recent visit to the Springs to take the B. & M. R. route of the Burlington system. The sleeping cars are comfortable and the officials of the road are polite and helpful.

This article has been written to call the attention of the medical profession to this new sanitarium in the Northwest. Quite frequently the physician is consulted by an invalid who has *worn out* many of the old health resorts of the country and is seeking some new El Dorado of health. Again, a patient has sought in many climates and various waters relief for his chronic rheumatism, or chronic bronchitis, or asthma, or consumption, and has been disappointed. I suggest that in such cases, as well as in others, the physician advise a visit to Hot Springs, S. D., often called "The Carlsbad of America." A comprehensive editorial article upon it may be found in the August, 1894, issue of *American Climates and Resorts*, written by the very competent climatologist, Dr. A. F. McKay.

My general impression of Hot Springs is that it combines many superior advantages as an all-the-year-round resort for those afflicted with chronic diseases and for those who wish to seek rest from the cares of business and relief from the "maddening crowd" of our large cities.

118 PLYMOUTH AVENUE.

